# Monitoring Early Infection Dynamics in Live *Spodoptera frugiperda* (Sf) 9 Cells via Optical Photothermal Infrared (OPTIR) Spectroscopy

**DOI:** 10.1021/acs.analchem.6c03567

**Published:** 2026-08-28

**Authors:** Shravan Raghunathan, Kristina Worch, Christoph Krafft, Volker Deckert, Antje Burse, Juergen Popp, Tanveer Ahmed Shaik

**Affiliations:** † 40096 Leibniz Institute of Photonic Technology, Member of Leibniz Health Technologies, Member of the Leibniz Centre for Photonics in Infection Research (LPI), Albert-Einstein-Straße 9, 07745 Jena, Germany; ‡ Abbe Center of Photonics (ACP), Friedrich Schiller University Jena, Member of the Leibniz Centre for Photonics in Infection Research (LPI), Albert-Einstein-Straße 6, 07745 Jena, Germany; § Department of Medical Engineering and Biotechnology, Ernst-Abbe-Hochschule, 59911 University of Applied Sciences, Carl-Zeiss-Promenade 2, 07745 Jena, Germany; ∥ Institute of Physical Chemistry (IPC), Friedrich Schiller University Jena, Member of the Leibniz Centre for Photonics in Infection Research (LPI), Helmholtzweg 4, 07743 Jena, Germany

## Abstract

Optical photothermal infrared (OPTIR) spectroscopy and imaging were combined with fluorescence microscopy to track infection progression. Baculovirus infection in *Spodoptera frugiperda* (Sf) 9 cells with mCherry as a fluorescence imaging reporter was analyzed 3, 6, 12, and 24 h postinfection (hpi). Cells were sandwiched between two CaF_2_ windows separated by a thin spacer, forming a sealed aqueous chamber that maintained hydration for 4–6 h, enabling IR spectral acquisition and imaging of living cells throughout. Fluorescence microscopy demonstrated a distinct onset of the mCherry reporter signal beginning 12 hpi. Early infection time points 3 and 6 hpi showed increased protein (amide II, 1545 cm^–1^) and asymmetric phosphate (1241 cm^–1^) bands, while 12 hpi, the symmetric phosphate (1087 cm^–1^) band profoundly reversed, consistent with nucleic acid changes associated with infection progression. The amide II to nucleic acid ratio (S_1545/1086_), obtained from discrete wavenumber imaging, tracked aggregate infection-associated protein changes (of which mCherry is a late-stage contributor), initially increasing 6 hpi, decreasing 12 hpi, and reaching its maximum value 24 hpi. The fluorescence reporter was absent at early stage infection (3 and 6 hpi), where IR spectroscopy and imaging successfully discriminated infected from uninfected cell populations. Fluorescence imaging validated IR measurements for later infection time points (12 and 24 hpi). Infected cells were classified from negative controls with balanced accuracies up to 90%. Overall, this approach identifies spectral signatures consistent with viral infection, providing a label-free method to discriminate infected populations from controls within their natural cellular environment.

## Graphical abstract

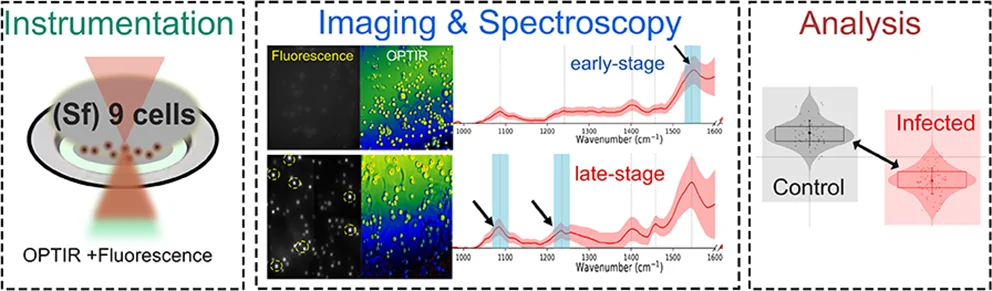

## Introduction

Vibrational spectroscopic techniques offer a powerful, label-free approach for biochemical assessment of body fluids,^1^ fixed and living cells,^2^ and tissue sections^3^ by probing molecular bond-specific information. Although state-of-the art label-based methods and assays are powerful tools for sensitive and specific biomarker detection,^4^ they require dedicated and often complex protocols, including time-consuming and elaborate sample preparation, cell fixation, permeabilization, and the need for specific labels like fluorescent dyes,^5^ antibodies, or genetically engineered cells.^6^ In contrast, infrared (IR) and Raman-based techniques can provide a rapid, noninvasive biochemical ″fingerprint″ of cellular composition and state, by directly analyzing the intrinsic molecular vibrations.

Earlier IR investigations of samples at a single cell level involved the study of mycoplasma in mammalian cells,^7^ malarian infections in erythrocytes,^8^ using Fourier transformed infrared spectroscopy (FTIR) by a focal plane array (FPA) detector. However, the spatial resolution limit due to the mid-IR radiation wavelengths, typically in the range of 3–8 μm, made it difficult to study individual cells. Furthermore, strong IR absorption bands of water can cause spectral overlap, thus IR spectra of dried cells are preferred over cells in an aqueous environment. Attenuated total reflection (ATR-FTIR) partially addressed these challenges, where an IR beam undergoes total internal reflection within a high refractive index (RI) crystal. This creates an evanescent wave that penetrates the lower RI sample by ca. half a wavelength, absorbing IR energy before the beam reflects, with its intensity indicating the sample’s absorption at a very shallow penetration depth (typically 0.5–5 μm). The use of ATR as a measurement modality with FTIR microscopy can increase spatial resolution by a factor equal to the ATR crystal’s refractive index.^9^ Furthermore, ATR combined with a synchrotron mid-IR source offers higher-SNR,^10^ micro-IR imaging capability^11,12^ and better chemical sensitivity.^13^

Although FTIR microspectroscopy and ATR-FTIR helped study single cells and infections at these levels, they were already pushing instrumental limits and capabilities. An indirect IR approach that overcomes limitations of traditional IR modalities is optical photothermal infrared (OPTIR) spectroscopy and imaging. Here, an IR excitation by a pulsed, tunable quantum cascade laser (QCL) is focused onto a sample that induces molecular absorption, i.e. vibration of biomolecules. This causes a modulated local sample heating. This heat-induced temperature change, which leads to changes in physical properties, such as linear expansion and refractive index, when the mid-IR wavelength is tuned, is referred to as the photothermal response or effect. This resultant photothermal response of the sample due to absorption can be probed by a visible laser. The visible probe is colinearly focused onto the same spot as the IR. The OPTIR signal is attained by registering the changes in sample expansion^14^ and/or refractive index modulation,^15,16^ by the optical detection of the probe beam. Advantages of OPTIR include improved spatial resolution and local chemical sensitivity, as photothermal registration is only limited by the wavelength of the visible probe laser. Additionally, refractive index modulation contrast being the primary contributor to the OPTIR signal means that spectra and IR imaging of live cells in their aqueous environment can be performed. Analysis of live and fixed cells using OPTIR of pancreatic and breast cancer cell lines has been demonstrated.^17^ It was reported previously that cultivated leukemia and cancer cell lines could be studied in an aqueous buffer using a CaF_2_ sandwich configuration, using discrete high-speed wavenumber OPTIR imaging and spectroscopy, followed by a model developed using linear discriminant analysis.^18^ More importantly, infections at the single cell level using OPTIR have been previously demonstrated to study bacterial infections, combining IR and Raman as a multimodal approach^19^ and *Plasmodium falciparum*-infected human erythrocytes, as a comparison of OPTIR against other traditional IR modalities.^20^ Fluorescence microscopy has also been combined with OPTIR to provide labeled targeting of IR analysis and enhanced measurement sensitivity.^21,22^ Some novel attempts for disease screening using custom home-built OPTIR setups have also been showcased to differentiate between migratory cancer and healthy cells.^23^ Recently, OPTIR spectral and image acquisition in an aqueous buffer of live cells and organisms, by using IR active probes to monitor specific proteins and molecular pathways was successfully demonstrated.^24^

In this current work, a multimodal approach of OPTIR imaging and spectroscopy with fluorescence microscopy is applied to meticulously track the temporal progression of baculovirus infection in live *Spodoptera frugiperda* (Sf) 9 cells, at 3, 6, 12, and 24 h postinfection (hpi). A specialized CaF_2_ sandwich configuration, which maintains optimal cell viability within an aqueous buffer environment and stable hydration levels for four to 6 h was utilized. OPTIR spectra were directly collected from individual living cells within the aqueous buffer, successfully identifying crucial biomolecular signals despite the strong water absorption. The mCherry fluorescence signal confirmed the infection status and heterogeneity. This novel approach utilized principal component linear discriminant analysis (PCA–LDA) on cell spectra to effectively distinguish early infected states of cells from uninfected ones with high accuracy. IR imaging also revealed key dynamic shifts in molecular components such as proteins (1545 cm^–1^, amide II) and nucleic acids (1087 and 1241 cm^–1^, phosphate groups). This robust, label-free method provides high-resolution spectroscopic insights into changes in biomolecular composition within individual living cells, in their natural environment, thus representing a critical step toward advanced understanding and identification of infection mechanisms.

## Materials and Methods

### Sample

*Spodoptera frugiperda* (Sf) 9 cells (Leibniz Institute DSMZ, Braunschweig, Germany) were maintained in Insect-XPRESS medium (Lonza, Cologne, Germany) as described in detail in an earlier publication.^25^ A 10 mL cell suspension (1.0 × 10^6^ cells/mL) was infected with recombinant baculovirus (encoding mCherry^26^ driven by the polyhedrin promoter; generated via the Bac-to-Bac Baculovirus Expression System^27^) at a multiplicity of infection (MOI) of 2, based on titers calculated by plaque assay.^25^ Measurements took place at 3, 6, 12, and 24 h postinfection (hpi). In addition to IR measurements, cell number and viability (trypan blue exclusion), along with cell size and fluorescence, were quantified using a Countess 3 Automated Cell Counter (Thermo Fisher Scientific, Schwerte, Germany). Samples were run in duplicates.

The sample chamber system used to immobilize the cells in an aqueous medium was achieved by a CaF_2_ sandwich configuration. The geometry and placement of the two CaF_2_ slides (thickness 200 μm) in the holder are highlighted below in Figure 1a. First, the bottom CaF_2_ slide, containing a 200 μm thick spacer, was coated with a thin layer of Poly-d-Lysine (GibcoTM, Thermo Fisher Scientific, Waltham, MA, USA) by a process of passive adsorption coating. Then, a 25 μL volume (estimated by calculating the volume supported by a spacer of thickness 200 μm and inner diameter of 12 mm) of the cell suspension in media was added to the slide. After waiting for ∼ 5 min to enable cell adhesion, the top slide was added to create the sandwich. The entire system was inverted carefully and placed onto the holder. Finally, the sample system was sealed by placing a silicone sheet (thickness ∼ 1 mm, Grace Bio-Laboratories, Merck, Darmstadt, Germany) with a clear aperture of ∼ 10 mm and then loaded into the instrument for measurement. The silicone-sealed CaF_2_ sandwich configuration enabled ∼ 4–6 h of cell measurements without sample drying. A fresh CaF_2_ sandwich chamber was prepared for each time point (3, 6, 12, and 24 hpi) rather than tracking a single chamber across the full 24 h infection window. Cells were infected in bulk in the flask, and a subsample was transferred to a freshly prepared chamber shortly before each time-point’s measurement session.

**Figure 1:**
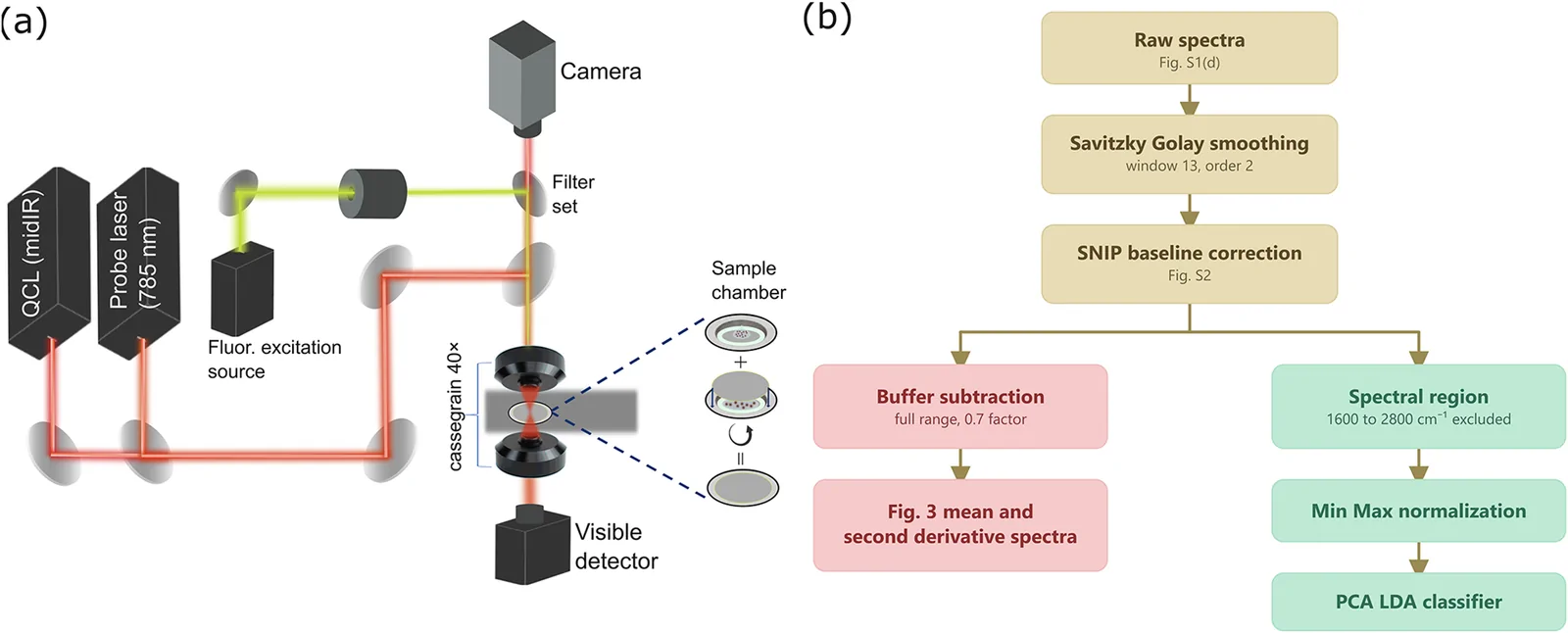
(a) Schematic diagram of the optical photothermal infrared (OPTIR) imaging, spectroscopy, and fluorescence imaging setup. A quantum cascade laser (QCL) mid-infrared pump and a 785 nm probe laser are simultaneously focused on the sample at the same spot. The OPTIR signal is detected using a visible photodetector. Brightfield and fluorescence images are captured by a camera, using a filter set consisting of excitation, dichroic, and emission filters. A custom-made sample chamber is used for the immobilization of cells in an aqueous medium, using a CaF_2_ sandwich configuration. Spectra and images are collected using two Cassegrain 40× (0.8 NA) objectives with similar specifications in transmission mode. (b) Overview of the spectral preprocessing and multivariate analysis workflow applied to single-cell OPTIR spectra, comprising Savitzky–Golay smoothing, SNIP baseline correction, removal of the 1600–2800 cm^–1^ interval, Min–Max normalization, and PCA–LDA classification with 10-fold cross-validation. Parallel branches indicate buffer subtraction (scaling factor of 0.7) used for only mean-spectrum visualization and second-derivative band assignment.

### Instrumentation and Data Acquisition

The OPTIR spectra, brightfield and fluorescence images were acquired with the mIRage-LS microscope, which uses a 785 nm probe laser (Photothermal Spectroscopy Corporation, Santa Barbara, CA, USA). A schematic diagram of the instrument is represented in Figure 1a below. The pump source is a four-module-pulsed, tunable quantum cascade mid-infrared laser (MIRcat QTTM, Daylight Solution, San Diego, CA, USA), which provides a spectral range from 973 to 1855, 1980 to 2390, and 2695–2999 cm^–1^, respectively. The pulse rate of the mid-IR laser was set at 100 kHz with a pulse width of 100 ns at a duty cycle of 1%. The OPTIR spectrum was acquired at an interval of 2 cm^–1^, in a high SNR mode with a sweep speed of 200 cm^–1^/s and a spectral resolution of 6.6 cm^–1^. Each OPTIR spectrum was an average of four scans acquired in the above-specified spectral range. OPTIR images were also recorded at discrete wavenumbers. For a 500 × 500 μm^2^ field of view (FOV, used for INF cells), the speed was set to 1000 μm/s, and for a 300 × 300 μm^2^ FOV (used for a subset of NC acquisitions), a speed of 600 μm/s. Images were recorded at a step size of 1 μm/pt. Both the OPTIR spectrum and image were acquired with the pump laser operating at 46% (2.13 mW) average power in the above-specified spectral range and the probe laser set to 6% (1.37 mW) power. Both spectra and images were collected by a 40× Cassegrain objective lens in transmission mode, and the detector gain was set at 2×. The fluorescence images were collected using a 50× objective lens and a filter set containing an mCherry/Red filter (AlexaFluor 594, Chroma Technology Corp., Rockingham, VT, USA), with LED excitation at 60% power and 0.5 s exposure time. These parameters were kept identical across all fluorescence acquisitions to enable direct intensity comparison across time points.

OPTIR spectra and images were recorded in a sequential repeating pattern, for an accurate representation of biochemical information from cells as follows. First, a region of interest was located within the sample system, and a larger FOV (700 × 700 μm^2^) brightfield image was collected using the 10× objective. Then, a fluorescence and brightfield mosaic (532 × 532 μm^2^) that falls within this FOV was acquired using the 50× objective. A single cell was then located, and an instrument-guided autofocus procedure was performed to bring the 40× Cassegrain objectives into focus. A 500 × 500 μm^2^ FOV was set for OPTIR image acquisition. Two discrete wavenumber images corresponding to protein (1545 cm^–1^) and nucleic acid (1086 cm^–1^) vibrations, respectively, were recorded in succession. A total of ∼ 18 min was required for the two discrete-wavenumber images. Following this, cells were located precisely using the protein (1545 cm^–1^) image and the spectra were recorded at four locations within each cell. This procedure enabled the acquisition of spatially colocated OPTIR spectra, OPTIR discrete wavenumber and fluorescence images. The sequence of spectral and image acquisition, along with the representation of raw, unprocessed data, is illustrated in Figure S1. Total working time per session comprising two discrete-wavenumber images (at 1545 and 1086 cm^–1^) and autofocused high-SNR OPTIR spectral acquisition from multiple cells at a time point is approximately 2 h, well within the 4–6-h hydration stability window of the chamber.

### Spectral and Image Processing

For each cell, four OPTIR spectra were acquired, with each spectrum comprising four coadded scans to enhance signal-to-noise ratio. The collected data were then imported into RAMANMETRIX^28^ for preprocessing and analysis (workflow summarized in Figure 1b). Preprocessing was performed in a sequential workflow. First, high-frequency noise was reduced using a Savitzky-Golay smoothing filter (window size: 13, polynomial order: 2, derivative order: 0). Second, the baseline was corrected using the statistics-sensitive nonlinear iterative peak-clipping (SNIP) algorithm with 40 iterations. Mean OPTIR spectrum after smoothing and baseline correction is shown in Figure S2. A detailed analysis of noise due to fringing effects is shown in Figure S3. Finally, the 1600–2800 cm^–1^ interval was removed from the smoothed and baseline-corrected spectra, as it comprises the residual water bending band (∼1640 cm^–1^) and the biologically uninformative cell-silent region (∼1800–2800 cm^–1^). The spectra were then Min–Max normalized.

The preprocessed spectra were interpreted in two ways. For multivariate classification, the smoothed, baseline-corrected, and normalized spectra, without prior buffer subtraction, were submitted to a supervised principal component–linear discriminant analysis (PCA–LDA) model (Figure 4). This involved reducing the dimensionality of the spectral data using the first six principal components (PCs), followed by linear discriminant analysis (LDA) to identify latent variables that best separate predefined classes. Principal components were retained until the cumulative explained variance ratio exceeded 90% of the total variance. The robustness and classification performance of the PCA–LDA model was evaluated using 10-fold cross-validation. The negative control (NC) groups at 3 and 6 h were combined for classification with the infected group (INF) at 3 hpi and 6 hpi, while the NC groups at 12 and 24 h were combined for classification with INF at 12 hpi and 24 hpi, respectively. The results, including classification scores, LD coefficients and balanced accuracies, were subsequently exported for visualization. For qualitative spectral inspection, the smoothed and baseline-corrected spectra were instead subjected to buffer subtraction using a fixed scaling factor of 0.7; these spectra were reserved exclusively for the mean-spectrum representations (Figure 3a–f) and second-derivative band assignment (Figure 3g–l) and were not used for classification. For aqueous buffer subtraction and second- derivative (polarity reversed) calculation of spectra, custom Python scripts utilizing the ‘*pandas*’ and ‘*numpy*’ libraries were used. For water background subtraction, a single uniform scaling factor of 0.7 was applied to the aqueous buffer reference spectra across all measurements. The value of 0.7 was determined empirically as the smallest factor that eliminated the residual water bending contribution around 1640 cm^–1^ without producing negative absorbance in adjacent regions and was then applied uniformly to preserve intersample comparability and avoid per-spectrum tuning. The CaF_2_ sandwich configuration was prepared identically for every time point and replicate, with a 200 μm spacer, 25 μL fill volume, Poly-d-Lysine coating, silicone seal, and consistent instrument alignment, so the aqueous path length and shape of the water contribution remained invariant across samples. The second derivative of OPTIR spectra was calculated using the Spectramap^29^ Python package with Savitzky-Golay filter parameters of (15, 2, 2). In total, OPTIR spectra were acquired from duplicates of NC and INF cells over four experimental days, to enhance the reproducibility and reliability of the spectral observations. Final single cell OPTIR spectral counts are listed in Table S1.

For the imaging analysis, all image processing and data extraction steps were automated using custom Python scripts, utilizing the ‘*scikit-image*’, ‘*OpenCV*’ and ‘*numpy*’ libraries. Data matrices from discrete wavenumber images, corresponding to the amide II protein band (1545 cm^–1^) and the nucleic acid phosphate band (1086 cm^–1^), were imported. To suppress background interference and isolate the cellular signal, a morphological white top-hat transform with a structural element radius of 25 pixels was applied to both images. The images were preprocessed using Contrast Limited Adaptive Histogram Equalization (CLAHE) to enhance edge definition. Cellular boundaries were determined based on the morphology of the protein channel (1545 cm^–1^). Subsequently, automated single-cell segmentation was performed using *Cellpose*,^30^ a deep-learning-based algorithm, using the ‘*cytoplasm*’ model with a characteristic diameter setting of 20 pixels. Identified regions smaller than 100 pixels were filtered out to exclude fragmentation artifacts. To further restrict the analysis to well-focused adherent cells, only the top 40% of segmented cells by mean 1545 cm^–1^ intensity was retained. The 40% threshold was chosen empirically to match the visibly in-focus adherent fraction, excluding out-of-focus and weakly signaling cells below a defined intensity floor. The same cell set was used for the 1087 cm^–1^ channel to preserve per-cell correspondence. Mean cell intensities were extracted for every individual cell mask from both the protein (1545 cm^–1^) and nucleic acid (1086 cm^–1^) images. As a last step, to observe trends in the relative signal contributions of these two vibrational modes, a fractional amide II to nucleic acid ratio, S_1545/1086_ was calculated for each cell using the following equation,

$$S_{1545/1086}\;(\mathrm{amide II to nucleic acid ratio})=\frac{1545\;{\mathrm{cm}}^{-1}}{1545\;{\mathrm{cm}}^{-1}+1086\;{\mathrm{cm}}^{-1}}$$

Per-cell ratios from the two biological replicates (acquired on separate days from independent cell cultures and virus inoculations) were pooled per class (NC and INF at each time point) prior to statistical testing, matching the pooling strategy used for the PCA–LDA classification. Statistical analysis was performed on the cell-derived fractional ratio using the Python scientific stack (‘*SciPy*’ and ‘*scikit-posthocs*’). Due to the non-Gaussian distribution and unequal sample sizes of the data, the nonparametric Kruskal–Wallis H-test was used to evaluate global differences. Pairwise comparisons between the control (NC) and infected (INF) groups were conducted using Dunn’s posthoc test with Bonferroni correction. This methodology was selected for its robustness in handling skewed distributions and controlling the group-wise error rate.

## Results and Discussion

### Fluorescence Imaging of Sf9 Cells

We used fluorescence images from the mCherry/red (MCHC) channel as a baseline to compare various infection time points. Images were captured from both the negative control and infected cells at 3 hpi, 6 hpi, 12 hpi, and 24 hpi, as shown in Figure 2.

**Figure 2:**
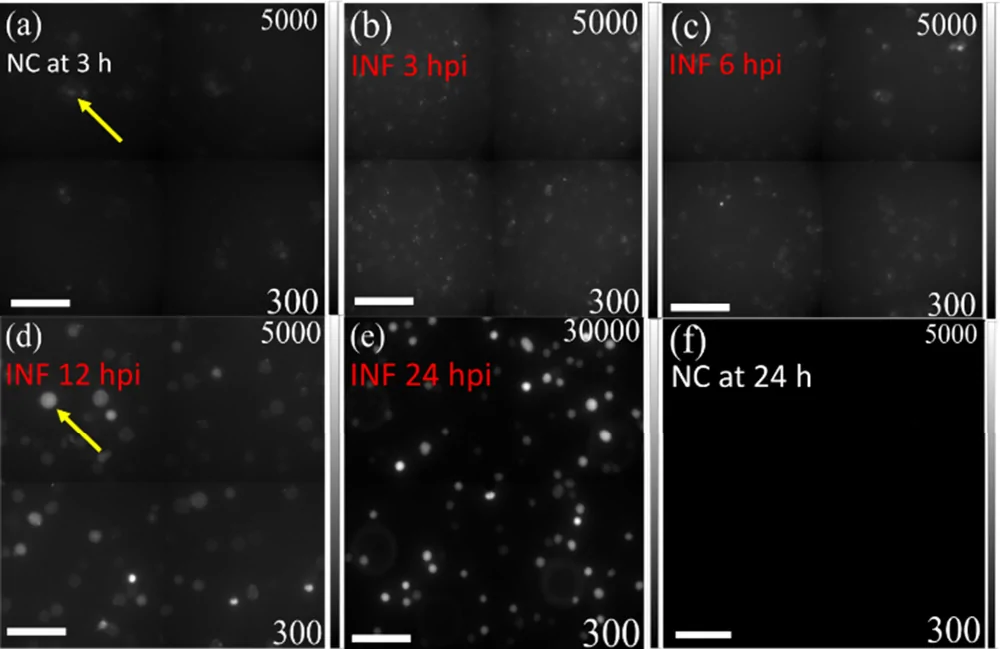
Fluorescence images of *Spodoptera frugiperda* (Sf) 9 cells. Negative control (NC) is shown at (a) 3 and (f) 24 h, while infected cells (INF) are shown (b) 3 h postinfection (hpi), (c) 6 hpi, (d) 12 hpi, and (e) 24 hpi. Scale bars in panels a–f are 100 μm.

The baculovirus genome was genetically modified to express mCherry as a fluorescent reporter, providing a visible indicator for tracking the presence of infection in Sf9 cells. The Sf9 cells at early infection time points (3 hpi, Figure 2b and 6 hpi, Figure 2c) and the respective negative controls (3 h, Figure 2a and 24 h, Figure 2f) were not fluorescent, confirming the absence of detectable mCherry production. At 12 hpi (Figure 2d), the onset of fluorescence in individual cells marks the transition to the very-late phase of infection, at which the polyhedrin promoter driving mCherry expression fully activates. Because the polyhedrin promoter is a late-phase viral promoter, mCherry fluorescence should be interpreted as an indicator of infection progression to the late-phase, rather than as a marker of infection onset. At 24 hpi (Figure 2e), the Sf9 cells exhibit the strongest fluorescence. Notably, between 12 hpi and 24 hpi, the fluorescence intensity increases ∼ 6-fold (from the intensity scales in Figure 2d and 2e).

The fluorescence images yield critical observations to categorize baculovirus infection levels as early stage (3 hpi and 6 hpi) and late stage (12 hpi and 24 hpi). Additionally, the heterogeneity of fluorescent intensities in individual cells indicates that infection does not begin simultaneously in all living Sf9 cells. This heterogeneity provides crucial insights for spectral and chemometric evaluations to be performed. While fluorescence imaging detects late-stage infection, the absence of mCherry signal at early time points (3 hpi, 6 hpi) precludes infected cell identification. Complementary infrared (IR) spectroscopy addresses this limitation via label-free biomolecular detection. As discussed below, IR spectral and imaging analysis is explored to differentiate infected cells from negative controls even during prefluorescent stages, capturing biochemical signatures undetectable by fluorescence microscopy.

### OPTIR Spectra of Live Sf9 Cells in an Aqueous Buffer

Figure 3. shows the mean and standard deviation of buffer/water subtracted OPTIR cell spectra and their corresponding second derivatives (polarity reversed), obtained from NC and infected Sf9 cells at 3 hpi, 6 hpi, 12 hpi, and 24 hpi, respectively. The corresponding spectra with buffer background retained are shown in Figure S2. Mean OPTIR spectra (Figure 3 a-f) showed consistent IR bands at 1545, 1459, 1401, and 1087 cm^–1^ in the fingerprint region and at 2855, 2877, 2923, and 2961 cm^–1^ in the high-wavenumber region.^17,18^ The standard deviations in the OPTIR spectra represent cell-to-cell variation after normalization. The amide II band at 1545 cm^–1^ is due to the in-plane bending of the N–H group and C–N stretching mode vibrations. The broad O–H bending band vibration of water was detected at 1641 cm^–1^ (Figure S2) and overlaps with the amide I band near 1660 cm^–1^. The vibration modes at 1459 and 1401 cm^–1^ are CH_2_ deformation of proteins and COO^–^, respectively. Specifically, the band at 1087 cm^–1^ can be assigned to the symmetric stretching modes of PO_2_^–^, found in the nucleic acids of ribonucleic acid (RNA)/ deoxyribonucleic acid (DNA).

**Figure 3:**
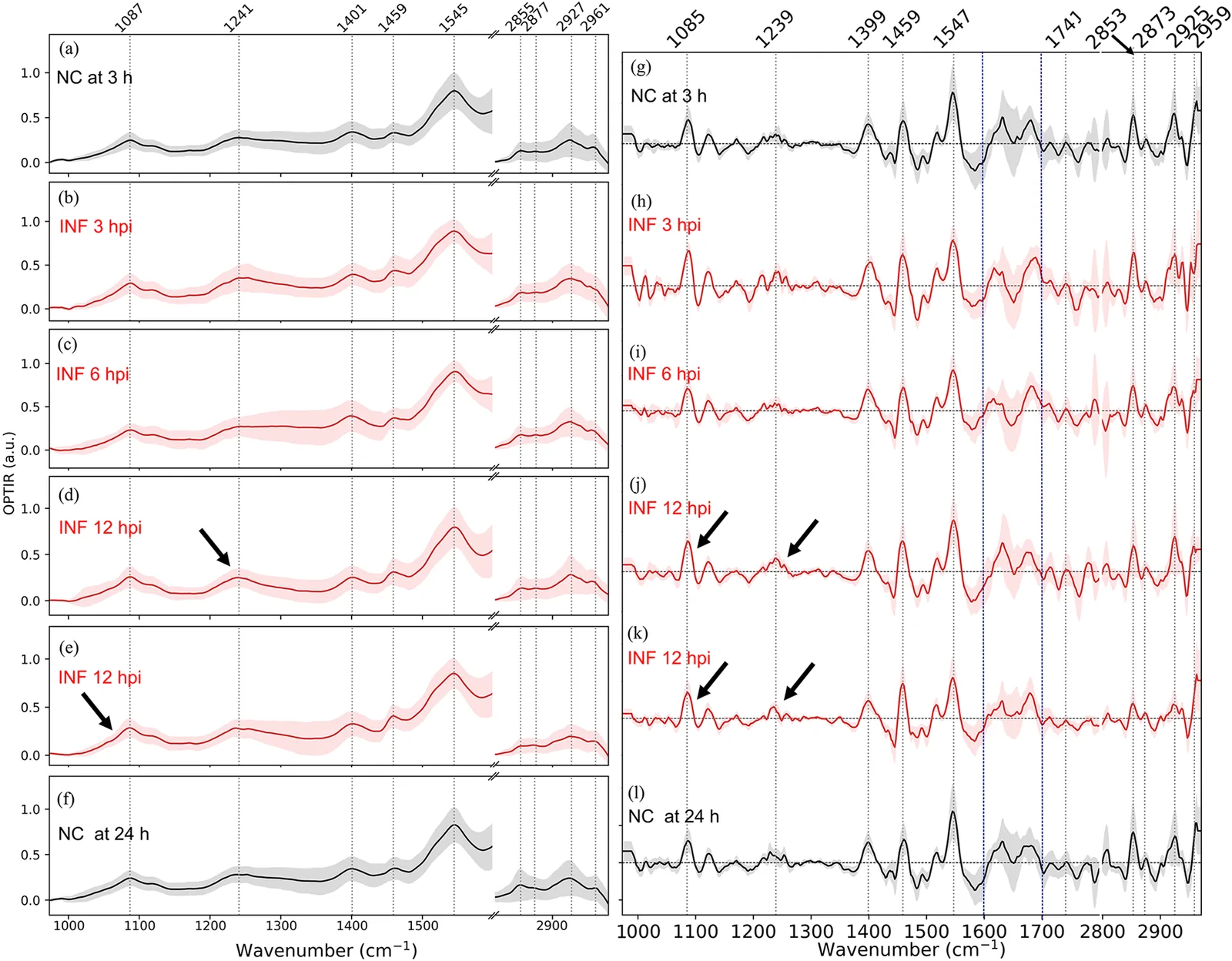
OPTIR spectra of *Spodoptera frugiperda* (Sf) 9 cells. Mean and standard deviation (mean ± SD) OPTIR spectra, with buffer subtraction (left column) and negative second derivative (right column) obtained from negative control (NC) and infected Sf9 cells (INF) at different time points. Negative control cells are shown at (a and g) 3 and (f and l) 24 h and infected cells (b and h) 3 h postinfection (hpi), (c and i) 6 hpi, (d and j) 12 hpi, and (e and k) 24 hpi. Panels a–f show buffer-subtracted spectra up to 1600 cm^–1^ and continue onto the high-wavenumber region; panels g–l show the corresponding second derivatives across the full range. The blue dashed lines in panels g–l mark the 1600–1690 cm^–1^ amide I region, which is obscured by residual water absorption in the aqueous chamber and is therefore excluded from downstream chemometric analysis.

In addition to the characteristic bands mentioned previously, the subtraction process revealed the band at 1241 cm^–1^, due to antisymmetric stretching modes of PO_2_^–^, found in the nucleic acids of RNA/DNA. This band was weak in the spectra with buffer background retained (Figure S2) and is crucial for further discussions. In the high wavenumber region, the symmetric stretching of CH_2_ (lipids) at 2855 cm^–1^ and an antisymmetric stretching of the same functional group at 2927 cm^–1^ can be identified. Additionally, a significant antisymmetric stretching band of the CH_3_ (also associated with lipid, fatty acids) group at 2961 cm^–1^ was detected.

Furthermore, the second derivatives (polarity retained with original spectra) were calculated for the individual OPTIR cell spectra at all the infection time points and the negative controls (Figure 3g-l). Maxima in the second derivative (negative) resolved more bands that overlapped in the broad envelope and were not evident. The amide II at 1547 cm^–1^, vibration modes at 1459 and 1399 cm^–1^ attributed to CH_2_ deformation of proteins and COO^–^ respectively, are consistent with the mean spectra as expected. Additionally, the second-derivative calculation successfully resolved the band at 2873 cm^–1^ due to the symmetric (lipid) stretching band of the (CH_3_) group, along with other bands at 2853, 2925, and 2959 cm^–1^ in the high-wavenumber region. The protein features in the amide I region, expected in the region between 1600 and 1690 cm^–1^, could not be resolved. The reason for this is the large column of aqueous buffer between the CaF_2_ substrates, which is essential to maintain live-cell viability and hydration, preserve cell adhesion on the Poly-d-Lysine-coated substrate, and enable OPTIR imaging of cells in their native hydrated state. This elucidates the choice of truncating the spectra at 1600 cm^–1^ for further PCA–LDA analysis. Detailed band assignments are summarized in Table S2.

Sample acquisition involved a 25 μL cell-in-buffer suspension within a 200 μm spacer. The early stage INF time points at 3 hpi and 6 hpi were particularly challenging for preparation and execution. Unlike other IR measurements, refilling the aqueous buffer between acquisitions was impossible; the dynamic nature of infection meant any tampering with the chamber could alter local aqueous levels, disrupt cell adhesion, or introduce microbial contamination. Such disruptions would alter single-cell dynamics and introduce unwanted heterogeneity. To minimize potential background variance, all OPTIR measurements were performed under a uniform aqueous environment across all control and infected samples. This methodological consistency through standardized CaF_2_ chamber preparation ensures that the background contributions remain consistent throughout the data set. Crucially, our chemometric analysis relies on reproducible relative spectral changes rather than absolute intensity differences, effectively mitigating the impact of baseline fluctuations.

### Classification of Infected Sf9 Cells

To robustly discriminate between NC and INF cells based on their OPTIR spectral fingerprints across a 24-h infection period, principal component analysis (PCA), providing dimensionality reduction, followed by linear discriminant analysis (LDA) was applied. Figure 4 shows the result of PCA–LDA analysis. The left panels in Figure 4 a-d display violin plots illustrating the distribution of linear discriminant (LD) scores of individual cells. The scores are projected onto their optimal linear discriminant axis, effectively separating NC from INF at all time-points. The balanced accuracy for each time-point discriminant is indicated. The linear discriminants shown in the right panels in Figure 4 a-d′, represent the corresponding LDA coefficients, revealing the spectral wavenumbers that most significantly contribute to the observed separation. Negative bands in the LD coefficient indicate a higher contribution to the model from INF cells relative to NC, while positive bands suggest otherwise.

**Figure 4:**
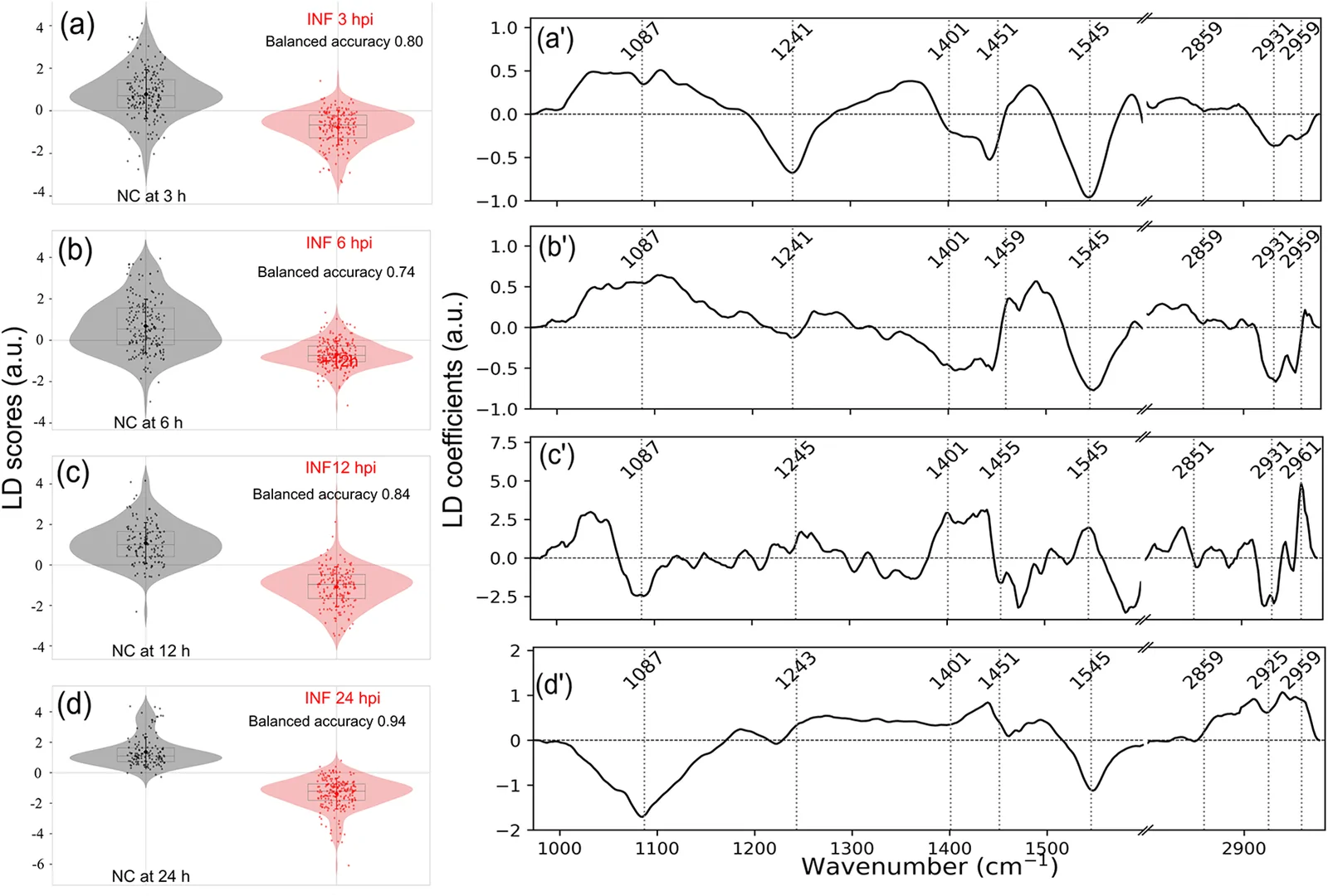
Principal component linear discriminant (PCA–LDA) analysis results. (a–d) Score plots and (a′–d′) corresponding LD coefficients for negative control (NC) and infected *Spodoptera frugiperda* (Sf) 9 cells (INF) (a) 3, (b) 6, (c) 12, and (d) 24 hpi. Each panel is an independent PCA–LDA classifier, so score and coefficient magnitudes are not comparable across panels. Spectral counts per classifier are listed in Table S1.

At the early stages of infection, 3 hpi and 6 hpi, discrimination was achieved with balanced accuracies of 0.80 (Figure 4a) and 0.74 (Figure 4b), respectively. The LD scores are distributed around the median ± SD values of 0.7 ± 1.2 for the NC (average for 3 and 6 h) and around −0.67 ± 0.8 and −0.7 ± 0.65 for the INF time-points, respectively. Based on the standard deviation of the scores, the points are clustered tightly for the 6 hpi compared to the 3 hpi, where they are spread more evenly. Despite a noticeable difference based on these balanced accuracies, they show a slight overlap, around the zero median value between NC and INF. At 12 hpi (Figure 4c), the population heterogeneity of infections is clear from the LDA median scores, centered around −0.96 ± 0.99, extending twice as compared to 3 hpi and 6 hpi. This indicates that multiple cells are at different stages of infection levels, with contributions from earlier stages and the time elapsed in between. The LD scores are distributed around 1.0 ± 1.0 for the NC at 12 h. The balanced accuracy for discrimination remained high at 0.84, showing successful separation of INF and NC cells. At 24 hpi (Figure 4d), the discrimination achieved the highest balanced accuracy of 0.94, demonstrating an established and highly distinct biochemical fingerprint of INF cells from NC. The LD median scores for INF cells at −1.2 ± 1.01 and NC at −1.1 ± 0.98 show the most tightly clustered distribution so far.

The LDA coefficients are plotted in the right panels of Figure 4. At the early stages of infection, 3 hpi (Figure 4a′) and 6 hpi (Figure 4b′), the positive coefficients of the symmetric stretching phosphate PO_2_^–^ band at 1087 cm^–1^, indicate that NC cells contribute positively to the model as compared to INF cells. The lipid-related CH_2_/CH_3_ bending modes experience distinct shifts over the observed time periods. At 3 hpi, these modes display a negative contribution at 1451 cm^–1^. However, by 6 hpi, this dynamic changes, as the contribution becomes positive and moves to a slightly higher position at 1459 cm^–1^. In contrast, the infected cells demonstrate higher negative coefficients when compared to the negative control across several other molecular regions. Specifically, these pronounced negative values are detected in the COO^–^ group at 1401 cm^–1^, throughout the C–H stretching region at both 2931 and 2959 cm^–1^, within the amide II band at 1545 cm^–1^, and along the phosphate PO_2_^–^ asymmetric stretch at 1241 cm^–1^. The LDA coefficients at 12 hpi (Figure 4c′) indicate significantly more pronounced and distinct biochemical differences between NC and INF cells. Here, the symmetric stretching phosphate 1087 cm^–1^ (PO_2_^–^) band, which exhibited strong positive coefficients at 3 hpi and 6 hpi, now displays a significant negative coefficient (from +0.5 to −2.5). This represents a critical reversal in its contribution to discrimination. In the context of infection biology, this time-point indicates a transition from early to late stage of infection, consistent with nucleic acid changes associated with the onset of baculoviral infection.^31,32^ The amide II band (1545 cm^–1^) reverses to a positive coefficient at 12 hpi. Although this is the time point at which mCherry first becomes traceable by fluorescence (Figure 2d), the positive coefficient indicates that the net amide II contribution at this stage is complex and not driven by mCherry alone. The CH_2_ bending (1455 cm^–1^) also contributes negatively to the model. Concomitantly, a positive coefficient is now observed for the 1245 cm^–1^ (asymmetric phosphate, PO_2_^–^) band. In addition, the COO^–^ symmetric stretch (1401 cm^–1^), the CH stretching region (2859, 2931, 2959 cm^–1^) also exhibit a notable, complex, and mostly positive contribution. At 24 hpi, LDA coefficients in Figure 4d′, show a clear shift from earlier time points. Infected cells at this stage exhibit even stronger negative coefficients at the phosphate PO_2_^–^ band at 1087 cm^–1^, continuing the trend as observed in 12 hpi. Similar to early infections (3 hpi and 6 hpi), the amide II band (1545 cm^–1^) again shows a negative contribution to the discriminant. This coincides with mCherry expression reaching its maximum at 24 hpi, while the nucleic acid changes due to infection also remain consistent. Although the sign of the amide II coefficient matches that at 3 and 6 hpi, the underlying contributors differ, with mCherry now a major component in addition to earlier protein changes. Conversely, positive coefficients persist in the lipid-related CH_2_/CH_3_ bending (1451 cm^–1^), asymmetric phosphate band at 1243 cm^–1^, CH stretching regions (2859, 2925, 2959 cm^–1^), and the COO^–^ band (1401 cm^–1^). All the bands mentioned here can be compared against the second derivatives (Figure 3g-l), calculated for the OPTIR spectra at all the infection time points.

Taken together, the LD analysis captures the transition from early to late stages of infection with precision. At early stages (3 and 6 hpi), discrimination relied on subtle but reproducible shifts in phosphate, lipid, and amide II contributions, yielding balanced accuracies of 0.80 and 0.74 despite the absence of any fluorescence reporter signal at these time points (Figure 2b,c). At late stages (12 and 24 hpi), the LD coefficients revealed pronounced biochemical reorganization, most notably the sign reversal of the symmetric phosphate band at 1087 cm^–1^, driving balanced accuracies up to 0.84 and 0.94, respectively. These late-stage classifications are independently validated by the onset and intensification of the mCherry fluorescence reporter (Figure 2d,e), confirming that the spectral signatures identified by LDA track genuine infection progression.

To ensure that these classifications reflect biology rather than model artifacts, the potential risk of overfitting inherent in high-dimensional spectral analysis was addressed using independent biological duplicates as internal validation. Spectra from the two independent biological replicates (acquired on separate days from independent cell cultures and virus inoculations) were pooled per class, and 10-fold cross-validation was applied to the pooled data so that each fold contained held-out cells drawn from both replicates. The consistent classification accuracy and stability of the LDA coefficients across these separate experimental runs support that the model captures genuine biochemical changes associated with infection, rather than stochastic noise or instrument drift.

### IR Imaging of Live Sf9 Cells in an Aqueous Buffer

IR images were acquired at 1545 cm^–1^ and 1087 cm^–1^, representing the biochemical distribution of the amide II and symmetric phosphate (PO_2_^–^) bands, respectively. The amide II band reflects infection-related protein production, whereas the phosphate band provides insight into changes in cellular DNA (nucleic acid) composition due to infection progression. IR images of NC and INF at 24 hpi acquired at 1545 cm^–1^ and 1087 cm^–1^ are shown in Figure 5a,b and d,e, respectively. Cells in both the NC and INF at 24 hpi are clearly resolved, despite certain artifacts due to underlying cells within the sample chamber. For the NC, multiple FOVs imaged at various infection time-points in parallel were combined. From these images, the single-cell fractional spectral ratio S_1545/1086_, reflecting the relative contribution of protein to the combined intensity of amide II (protein) and symmetric phosphate (DNA) stretching modes, was calculated. This ratio serves as a dynamic biomarker of the biochemical state of single cells during infection, tracking the interplay between infection-associated protein changes (including mCherry at late stages) and nucleic acid changes. Figure 5g illustrates the single cell ratios (S_1545/1086_) for NC and INF cells at all time points.

**Figure 5:**
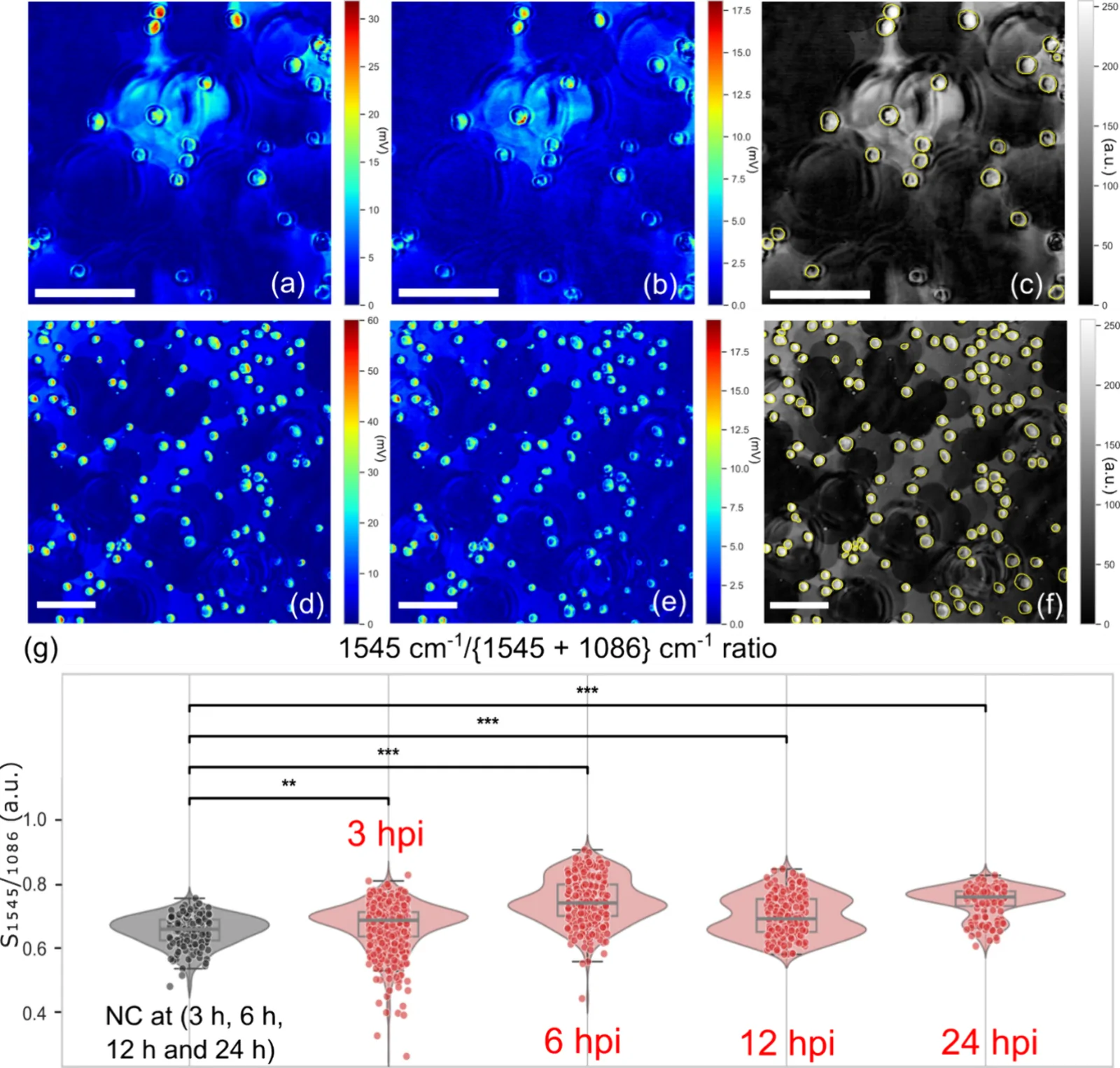
Discrete wavenumber imaging of *Spodoptera frugiperda* (Sf) 9 cells. (a) Negative control (NC) at 1545 cm^–1^, (b) NC at 1086 cm^–1^, (c) segmented NC cells using the 1545 cm^–1^ image, (d) 24 hpi infected cells (INF) at 1545 cm^–1^, (e) 24 hpi INF at 1086 cm^–1^, and (f) segmented 24 hpi INF cells using the 1545 cm^–1^ image. Scale bars in panels a–f are 100 μm. NC images (a–c) were acquired at 300 μm × 300 μm FOV, while INF images (d–f) were acquired at 500 μm × 500 μm FOV, both displayed at the same panel size; the scale bars correctly represent the physical scale in each case. (g) Violin plots of the amide II to nucleic acid ratio S_1545/1086_, from mean cell intensities of NC and INF 3, 6, 12, and 24 hpi. For the NC, cells from all time points were combined. Statistical significance was assessed using the nonparametric Kruskal–Wallis H-test for global comparisons across all groups. For specific pairwise comparisons against the control group (NC), Dunn’s post hoc test was applied with Bonferroni correction to adjust for multiple testing. Significance is reported as follows: *p < 0.05, **p < 0.01, ***p < 0.001, and *p* ≥ 0.05 is considered nonsignificant (ns). Total observations (cell counts) in each group: NC (*n* = 156), 3 hpi (*n* = 501), 6 hpi (*n* = 315), 12 hpi (*n* = 271), and 24 hpi (*n* = 144).

The median ± SD position of this ratio in infected cells demonstrates a progressive increase from 0.65 ± 0.05 for the NC through 0.67 ± 0.07 and 0.75 ± 0.07, respectively, for early infection (3 hpi and 6 hpi). This observed trend corresponds to an increase in overall protein content within INF cells, as supported by the LDA coefficients in Figure 4a,b. A negative protein 1545 cm^–1^ and positive symmetric phosphate (1086 cm^–1^) coefficient means a characteristic INF cell at early stages, would show a higher amide II (1545 cm^–1^) absorption relative to phosphate (1086 cm^–1^) compared to an NC cell. This highlights the advantage of IR vibrational imaging as compared to fluorescence microscopy. The separation of NC and INF at 3 hpi and 6 hpi was inconclusive by fluorescence imaging (Figure 2b,c), since early stages are associated with negligible mCherry production.

At 12 hpi, a distinct drop in the S_1545/1086_ median value to 0.7 ± 0.06, represents a nonmonotonic trend reflecting the complex interplay between mCherry expression and nucleic acid changes. This is consistent with the LD coefficients in Figure 4c′, where a reversal for the 1086 cm^–1^ band changing from positive to a strong negative contribution was first observed. Like the LD violin plots (Figure 4a-d) that showed the maximum dispersity, a doubly lobed distribution of the ratio around the median (0.7 ± 0.06) value can be observed here. This could be associated with measurements performed on two biological replicates, used to validate the study. While mCherry protein production (1545 cm^–1^) becomes detectable by 12 hpi, the rapid and substantial increase in denominator component (1086 cm^–1^) at this stage temporarily causes a relative decrease in S_1545/1086_, consistent with observed LD coefficients (Figure 4c′). At 24 hpi, where the most mCherry is produced and/or accumulated, the ratio reaches a maximum value of 0.74 ± 0.05, also consistent with the strong amide II negative LD coefficient in Figure 4d′. Statistical analysis of the S_1545/1086_ ratio across individual cells revealed a significant temporal progression of the infected cells (INF) as compared to the control group (NC). The Kruskal–Wallis H-test confirmed a highly significant global shift in cellular ratios across all time points (*p* < 0.001). Posthoc pairwise comparisons demonstrated a clear statistical separation beginning as early as 3 hpi (p = 0.00111, **) and reaching maximum significance from 6 hpi through 12 and 24 hpi (*p* < 0.001, ***).

Additionally, spectral observations from the LD analysis in Figure 4 support the changes observed here. It is worth noting that infection at MOI = 2 is expected to leave a small fraction of cells (∼14%, following Poisson statistics) initially unexposed within any imaged field of view, and cells were selected at random for IR measurement rather than prescreened for fluorescence. At late time points (12 and 24 hpi), mCherry positivity therefore provides only a lower bound on the true infection fraction, since weakly expressing cells may fall below the fluorescence detection limit and individual cells vary in how quickly they begin producing the reporter. The PCA–LDA framework therefore describes a population-level shift between exposed (INF) and unexposed (NC) cells rather than a per-cell diagnostic, which is consistent with the balanced accuracies observed at 3 and 6 hpi and with their rise at 12 and 24 hpi as mCherry becomes detectable. What matters for label-free discrimination is that the exposed and unexposed populations differ significantly, crossing a threshold beyond which samples containing infected cells become distinguishable from uninfected ones. The combination of the dynamic amide II to nucleic acid ratio S_1545/1086_, together with supportive analysis from the linear discriminants, precisely tracks the interplay between mCherry protein and nucleic acid changes, offering a qualitative and quantitative assessment of infection progression.

## Conclusion

Optical photothermal infrared (OPTIR) spectroscopy and imaging, combined with fluorescence imaging techniques, were used to study the progression of baculovirus infection in live *Spodoptera frugiperda* (Sf) 9 cells. This study examined infection at 3, 6, 12, and 24 h postinfection (hpi) to study the molecular makeup during infection. By utilizing standardized CaF_2_ chambers to ensure stable hydration, OPTIR and fluorescence successfully monitored infection-associated biochemical changes. Fluorescence imaging confirmed asynchronous transition to the late-phase of infection, with detectable mCherry expression appearing from 12 hpi onward, consistent with the polyhedrin promoter’s late-phase activation profile. In contrast, OPTIR enabled the discrimination between infected and control populations at 3 hpi and 6 hpi, before reporter signals were detectable. PCA–LDA facilitated this discrimination, achieving balanced accuracies of 74–94% across the infection cycle. The amide II/nucleic acid ratio obtained through discrete wavenumber imaging provided a label-free indicator of infection, offering complementary evidence of the protein shifts and the changes observed at 12 hpi, which are consistent with nucleic acid changes associated with infection progression.

By leveraging the unique capabilities of OPTIR and fluorescence, this study demonstrates a complementary platform for resolving cellular dynamics in near-native environments. Prior IR studies of infection have relied on fixed samples, parasitic (nonviral) models, or synchrotron sources, with limited combinations of live eukaryotic viral infection, aqueous-buffer transmission, single-cell resolution, and coregistered fluorescence on a single benchtop platform. OPTIR’s water-tolerant photothermal contrast, submicron effective resolution, and same-instrument fluorescence coregistration together enable this combination, with the baculovirus–Sf9 infection dynamics serving as biological validation of the analytical approach.

## Supplementary Material



## Data Availability

Data will be made available upon reasonable request.

## Notes

OPTIR spectra and discrete-wavenumber images alongside fluorescence imaging of infected Sf9 cells 12 hpi in an aqueous buffer (Figure S1), mean OPTIR spectra with an aqueous buffer (Figure S2), estimation of the CaF_2_ spacer thickness from the etaloning (fringing) effect (Figure S3), number of OPTIR spectra acquired per cell type and time point (Table S1), and IR band assignments used in the spectral interpretation (Table S2) (PDF)

## Abbreviations

IR: infrared
OPTIR: optical photothermal infrared
Sf9: *Spodoptera frugiperda* (Sf) 9
FOV: field-of-view
QCL: quantum cascade laser
FTIR: Fourier transformed infrared spectroscopy
FPA: focal plane array
ATR: attenuated total reflection
RI: refractive index
SNIP: statistics-sensitive nonlinear iterative peak-clipping
PCA–LDA: principal component analysis–linear discriminant analysis
PC: principal components
CLAHE: contrast limited adaptive histogram equalization
LD: linear discriminant
hpi: hours postinfection
NC: negative control
INF: infection
RNA: ribonucleic acid
