# Supplementary material for: Monitoring Early Infection Dynamics in Live Spodoptera frugiperda (Sf) 9 Cells via Optical Photothermal Infrared (OPTIR) Spectroscopy
Source: Anal Chem. 2026 Aug 28;98(36):26777–87. doi: 10.1021/acs.analchem.6c03567 (PMC13584692; doi:10.1021/acs.analchem.6c03567)
Supplement: Supplementary file 1 [file ac6c03567_si_001.pdf]

Supporting Information

Monitoring early infection dynamics in live *Spodoptera frugiperda* (Sf) 9 cells via optical photothermal infrared (OPTIR) spectroscopy

Shravan Raghunathan [a], Kristina Worch [c], Christoph Krafft [a], Volker Deckert [a,b,d], Antje Burse [c], Juergen Popp [a,b,d], and Tanveer Ahmed Shaik [a,b,d *]

a) Leibniz Institute of Photonic Technology, Member of Leibniz Health Technologies, Member of the Leibniz Centre for Photonics in Infection Research (LPI), Albert-Einstein-Straße 9, 07745 Jena, Germany

b) Abbe Center of Photonics (ACP), Friedrich Schiller University Jena, Member of the Leibniz Centre for Photonics in Infection Research (LPI), Jena, Germany, Albert-Einstein-Straße 6, 07745 Jena, Germany

c) Department of Medical Engineering and Biotechnology, Ernst-Abbe-Hochschule, University of Applied Sciences, Carl-Zeiss-Promenade 2, 07745 Jena, Germany

d) Institute of Physical Chemistry (IPC), Friedrich Schiller University Jena, Member of the Leibniz Centre for Photonics in Infection Research (LPI), Helmholtzweg 4, 07743 Jena, Germany

*Corresponding author

Table of Contents

Figure S1. OPTIR spectra and imaging (fluorescence and discrete wavenumber) of infected *Spodoptera frugiperda* (Sf) 9 cells at 12 h post-infection in aqueous buffer…........................... S3

Figure S2. Mean OPTIR spectra with buffer.……………………………………………… S4

Figure S3. CaF_2 spacer estimation from etaloning (fringing) effect………………………. S5

Table S1. OPTIR spectral counts of cell types…………………………………………….. S6

Table S2. IR band assignments………………………... S7

References…………………………...S8

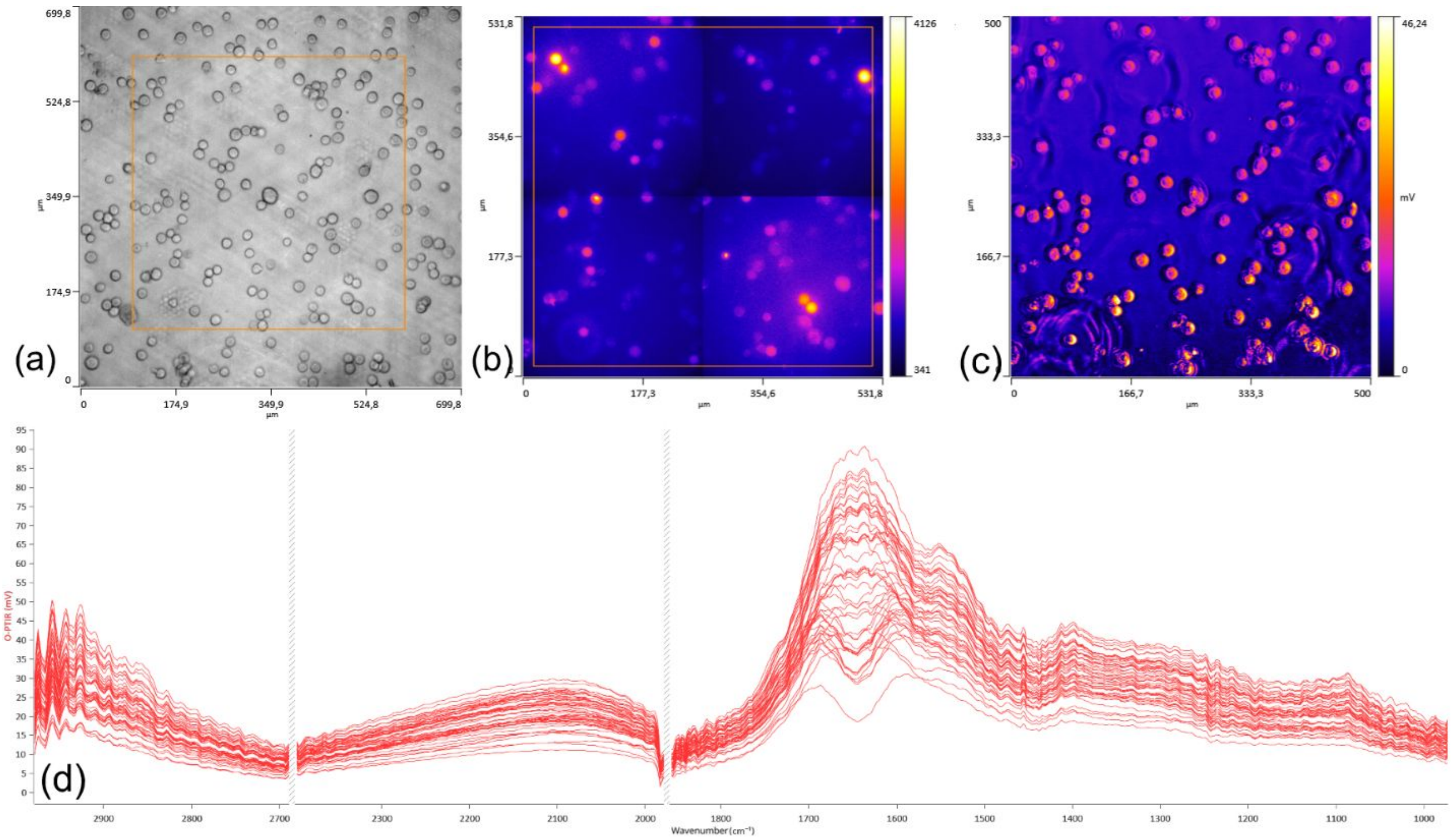

Figure S1. OPTIR spectra and imaging (fluorescence and discrete wavenumber) of infected *Spodoptera frugiperda* (Sf) 9 cells at 12 hours post-infection in aqueous buffer. (a) Microscope image of cells obtained using a 10× objective. (b) Fluorescence image (2×2 mosaic) of cells using the MCHC channel by a 50× objective (c) IR image at amide II (1545 cm^{-1}), field-of-view indicated in (a) and (b); (d) multiple OPTIR spectra acquired from cells in the field of view. Visible fringing effect confirmed by calculations in Fig. S3 below.

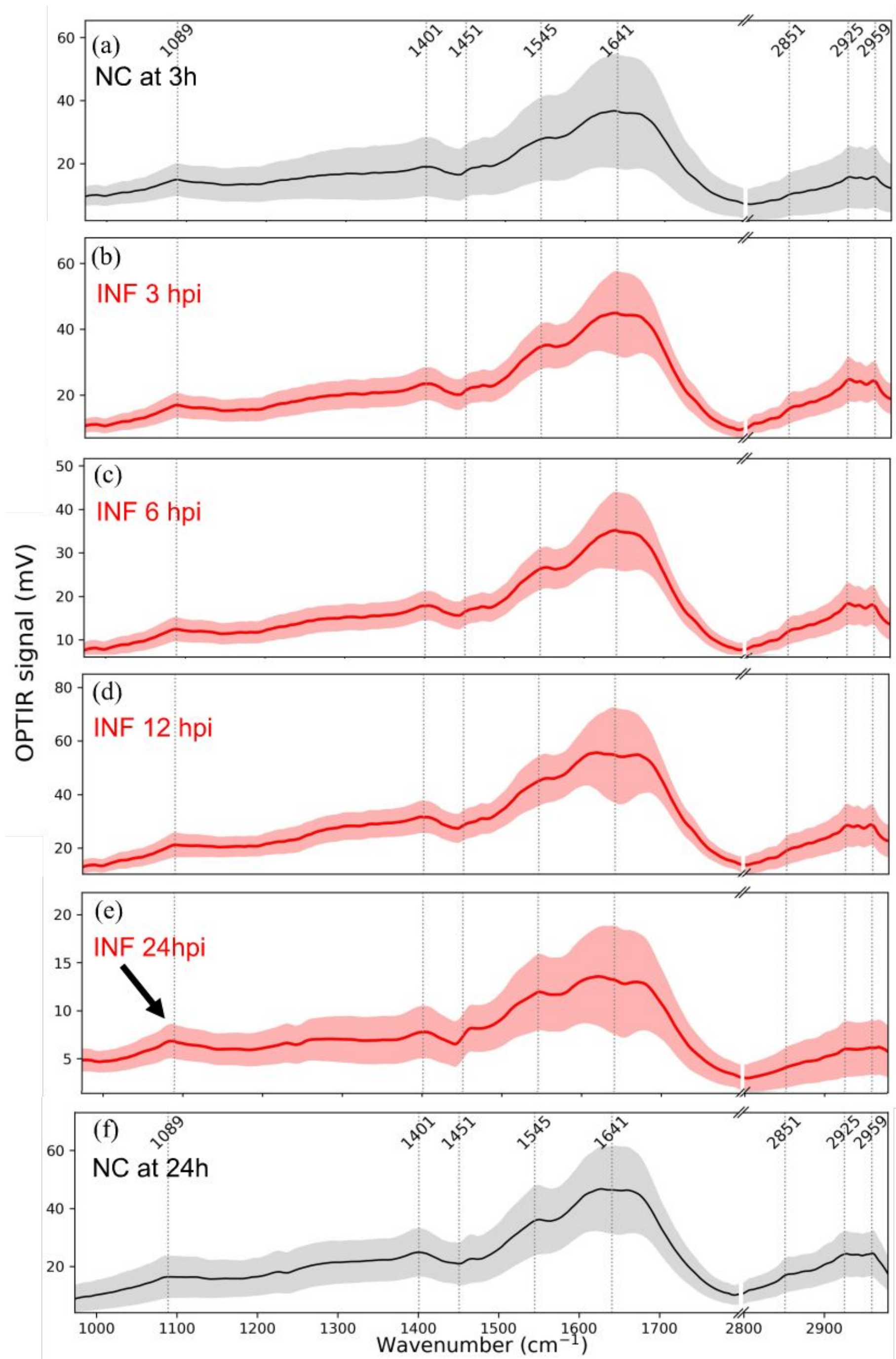

Figure S2. Mean and standard deviation (mean ± SD) OPTIR spectra obtained from negative control (NC) and infected (INF) *Spodoptera frugiperda* (Sf) 9 cells (with buffer) at different time

points. Negative control (black) cells are shown at (a) 3 h and (f) 24 h; infected (red) cells at (b) 3 hours post-infection (hpi); (c) 6 hpi; (d) 12 hpi; and (e) 24 hpi.

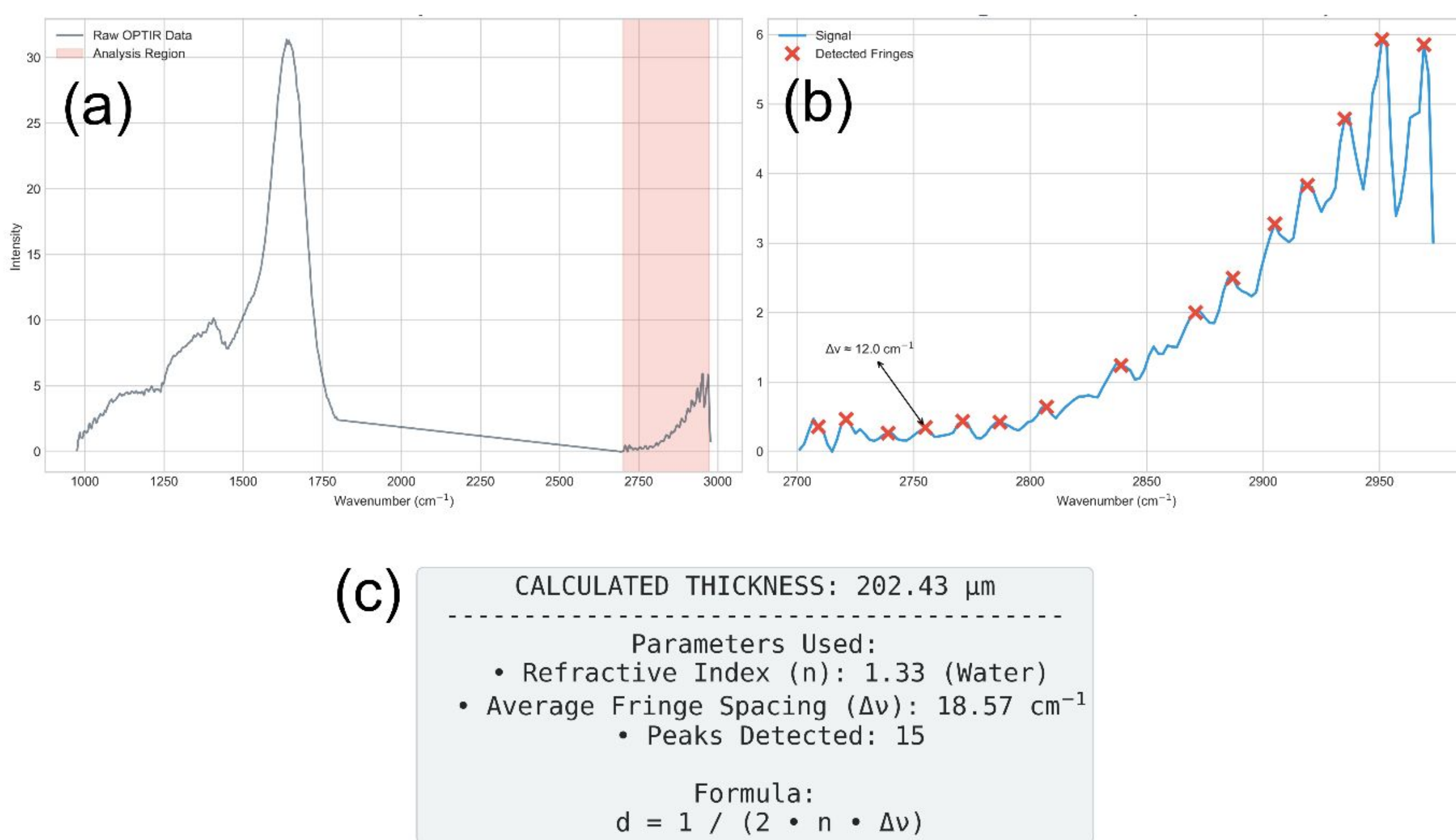

Figure S3. CaF_2 spacer estimation from etaloning (fringing) effect. (a) Raw, unprocessed (baseline-compensated) spectrum of an aqueous buffer. (b) standing wave patterns (fringing/etalon effect) in the high-wavenumber region (2700 – 2979 cm^{-1}), identified using the Python '*SciPy*' library (c) evaluation of effective sample thickness based on physical parameters and resonance condition.

Table. S1. OPTIR spectral counts of cell types.

**Cell type**	**Before buffer subtraction**	**After buffer subtraction**
**NC at 3 + 6 h**	185	102
**NC at 12 + 24 h**	132	122
**INF 3 hpi**	177	131
**INF 6 hpi**	221	149
**INF 12 hpi**	177	99
**INF 24 hpi**	212	151

Number of OPTIR spectra obtained from negative control (NC) and infected (INF) *Spodoptera frugiperda* (Sf) 9 cells at 3, 6, 12 and 24 hours post-infection (hpi). Values shown are for both before (for PCA-LDA analysis) and after (for Mean spectra and second derivative visualization) buffer subtraction.

Table S2. IR band assignments.

**Band positions (cm^{-1})**	**Band assignment**
**1085 / 1086 / 1087**	$\nu_s(PO_2-)$, Nucleic Acids (DNA/RNA)
**1239 / 1241 / 1243 / 1245**	$\nu_{as}(PO_2-)$, Nucleic Acids (DNA/RNA)
**1400 / 1401**	$\nu_s(COO^-)$, Proteins
**1451 / 1455 / 1459**	$\delta(CH_2)$/ $\delta(CH_2,CH_3)$, Proteins/ Lipids
**1545 / 1547**	amide II, ($\delta(N-H)+\nu(C-N)$), Proteins
**1641**	$\delta(O-H)$, Water (aqueous buffer)
**1741**	$\nu(C=O)$, Lipids
**2853 / 2855 / 2859**	$\nu_s(CH_2)$, Lipids

**2873**	$\nu_s(CH_3)$, Lipids / Proteins
**2923 / 2925 / 2931**	$\nu_{as}(CH_3)$, Lipids / Fatty Acids
**2959 / 2961**	$\nu_{as}(CH_3)$ / $\nu_{as}(CH_2)$, Lipids / Proteins

Band assignments of cells in OPTIR spectra, second derivatives and linear discriminants (LD coefficients)[1,2].

References

1. Shaik, T. A., Ramoji, A., Milis, N., Popp, J. & Krafft, C. Optical photothermal infrared spectroscopy and discrete wavenumber imaging for high content screening of single cells. *Analyst* 148, 5627–5635 (2023).

2. Gardner, P., Spadea, A., Denbigh, J., Lawrence, M. J. & Kansiz, M. Analysis of fixed and live single cells using optical photothermal infrared with concomitant Raman spectroscopy. *Anal. Chem.* 93, 3938–3950 (2021).
